# Supplementary material for: Shape analysis of the human association pathways
Source: Neuroimage. Author manuscript; Available in PMC 2021 Jan 1. (PMC7775618; doi:10.1016/j.neuroimage.2020.117329)
Supplement: 1 [file NIHMS1657556-supplement-1.docx]

**Subject ID in HCP test-retest dataset:**

103818 105923 111312 114823 115320 122317 125525 130518 135528 137128 139839 143325 144226 146129 149337 149741 151526 158035 169343 172332 175439 177746 185442 187547 192439 194140 195041 200109 200614 204521 250427 287248 341834 433839 562345 599671 601127 627549 660951 783462 859671 861456 877168 917255 917255
